# Violent aggression predicted by multiple pre-adult environmental hits

**DOI:** 10.1038/s41380-018-0043-3

**Published:** 2018-05-24

**Authors:** Marina Mitjans, Jan Seidel, Martin Begemann, Fabian Bockhop, Jorge Moya-Higueras, Vikas Bansal, Janina Wesolowski, Anna Seelbach, Manuel Ignacio Ibáñez, Fatka Kovacevic, Oguzhan Duvar, Lourdes Fañanás, Hannah-Ulrike Wolf, Generós Ortet, Peter Zwanzger, Verena Klein, Ina Lange, Andreas Tänzer, Manuela Dudeck, Lars Penke, Ludger Tebartz van Elst, Robert A. Bittner, Richard Schmidmeier, Roland Freese, Rüdiger Müller-Isberner, Jens Wiltfang, Thomas Bliesener, Stefan Bonn, Luise Poustka, Jürgen L. Müller, Bárbara Arias, Hannelore Ehrenreich

**Affiliations:** 1 Clinical Neuroscience, Max Planck Institute of Experimental Medicine, Göttingen, Germany; 2 DFG Research Center for Nanoscale Microscopy and Molecular Physiology of the Brain (CNMPB), Göttingen, Germany; 3 Instituto de Salud Carlos III, Centro de Investigación Biomédica en Red de Salud Mental (CIBERSAM), Madrid, Spain; 4 Department of Psychiatry & Psychotherapy, University of Göttingen, Göttingen, Germany; 5 Department of Psychology, Faculty of Education, Psychology and Social Work, University of Lleida, Lleida, Spain; 6 Center for Molecular Neurobiology, Institute of Medical Systems Biology, University Clinic Hamburg-Eppendorf, Hamburg, Germany; 7 Department of Basic and Clinical Psychology and Psychobiology, Universitat Jaume I, Castelló, Spain; 8 Departament Biologia Evolutiva, Ecologia i Ciències Ambientals, Facultat de Biologia and Institut de Biomedicina (IBUB), Universitat de Barcelona, Barcelona, Spain; 9 KBO-Inn-Salzach-Klinikum, Gabersee, Wasserburg am Inn Germany; 10 KBO-Isar-Amper-Klinikum, Taufkirchen (Vils), Germany; 11 Competence Center for Forensic Psychiatry, Lower Saxony, MRV Moringen Germany; 12 Department of Forensic Psychiatry & Psychotherapy, KRH, Wunstorf, Germany; 13 Forensic Psychiatry and Psychotherapy, University of Ulm, Ulm, Germany; 14 Institute of Psychology, University of Göttingen, Göttingen, Germany; 15 Department of Psychiatry & Psychotherapy, University of Freiburg, Freiburg, Germany; 16 Department of Psychiatry & Psychotherapy, University of Frankfurt, Frankfurt, Germany; 17 Vitos Forensic Psychiatric Hospital, Haina, Germany; 18 Criminological Research Institute of Lower Saxony, Hannover, Germany; 19 Department of Child and Adolescent Psychiatry & Psychotherapy, University of Göttingen, Göttingen, Germany; 20 Asklepios Hospital for Forensic Psychiatry & Psychotherapy, Göttingen, Germany

**Keywords:** Biomarkers, Neuroscience, Psychiatric disorders

## Abstract

Early exposure to negative environmental impact shapes individual behavior and potentially contributes to any mental disease. We reported previously that accumulated environmental risk markedly decreases age at schizophrenia onset. Follow-up of matched extreme group individuals (≤1 vs. ≥3 risks) unexpectedly revealed that high-risk subjects had >5 times greater probability of forensic hospitalization. In line with longstanding sociological theories, we hypothesized that risk accumulation before adulthood induces violent aggression and criminal conduct, independent of mental illness. We determined in 6 independent cohorts (4 schizophrenia and 2 general population samples) pre-adult risk exposure, comprising urbanicity, migration, physical and sexual abuse as primary, and cannabis or alcohol as secondary hits. All single hits by themselves were marginally associated with higher violent aggression. Most strikingly, however, their accumulation strongly predicted violent aggression (odds ratio 10.5). An epigenome-wide association scan to detect differential methylation of blood-derived DNA of selected extreme group individuals yielded overall negative results. Conversely, determination in peripheral blood mononuclear cells of histone-deacetylase1 mRNA as ‘umbrella mediator’ of epigenetic processes revealed an increase in the high-risk group, suggesting lasting epigenetic alterations. Together, we provide sound evidence of a disease-independent unfortunate relationship between well-defined pre-adult environmental hits and violent aggression, calling for more efficient prevention.

## Introduction

Early exposure to external risk factors like childhood maltreatment, sexual abuse or head trauma, but also living in urban environment or migration from other countries and cultures, have long been known or suspected to exert adverse effects on individual development and socioeconomic functioning. Moreover, these environmental risk factors seem to contribute to abnormal behavior and to severity and onset of mental illness [1–11], even though different risk factors may have different impact, dependent on the particular neuropsychiatric disease in focus. On top of these ‘primary’ factors, that are rather inevitable for the affected, ‘secondary’, avoidable risks add to the negative individual and societal outcome, namely cannabis and alcohol abuse [1, 11–16].

Adverse experiences in adulthood, like exposure to violence, traumatic brain injury, or substance intoxication, can act as single triggers to increase the short-term risk of violence in mentally ill individuals as much as in control subjects [16, 17]. However, comprehensive studies, including large numbers of individuals and replication cohorts, on pre-adult accumulation of environmental risk factors and their long-term consequences on human behavior do not exist. In a recent report, we showed that accumulation of environmental risks leads to a nearly 10-year earlier schizophrenia onset, demonstrating the substantial impact of the environment on mental disease, which by far outlasted any common genetic effects [18]. To search for epigenetic signatures in blood of carefully matched extreme group subjects of this previous study (with ≤1 vs. ≥3 risk factors) we had to re-contact them. This re-contact led to the unforeseen observation that high-risk subjects had > 5 times higher probability to be hospitalized in forensic units compared to low-risk subjects.

This finding stimulated the present work: Having the longstanding concepts of sociologists and criminologists in mind, we hypothesized that early accumulation of environmental risk factors would lead to increased violent aggression and social rule-breaking in affected individuals, independent of any mental illness. To test this hypothesis, we explored environmental risk before the age of 18 years in 4 schizophrenia samples of the GRAS (Göttingen Research Association for Schizophrenia) data collection [19, 20]. Likewise, risk factors were assessed as available in 2 general population samples. In all cohorts, accumulation of pre-adult environmental hits was highly significantly associated with lifetime conviction for violent acts or high psychopathy and aggression-hostility scores as proxies of violent aggression and rule-breaking. As a first small hint of epigenetic alterations in our high-risk subjects, histone-deacetylase1 (*HDAC1*) mRNA was found increased in peripheral blood mononuclear cells (PBMC).

## Methods

### Subjects

#### Schizophrenia

Ethics Committees of Georg-August-University, Göttingen, and participating centers across Germany approved the GRAS study, complying with the Helsinki Declaration. All patients (and/or legal representatives) gave written informed consent. GRAS data collection-I (2005–2010) [19, 20] and -II (2013–2016) consist of schizophrenic and schizoaffective subjects, assigned to: (1) male discovery sample (*N* = 134 extreme group individuals with ≤1 or ≥3 risk factors, selected/matched from our previous study [18]); (2) male GRAS-I (*N* = 606); (3) male GRAS-II (*N* = 320); (4) female GRAS-I and -II cohorts (*N* = 503).

#### General population

Replication samples IV (*N* = 336) and V (*N* = 229) consist of individuals from the Spanish general population, recruited from the Jaume I University in Castelló and drawn from the third wave of an ongoing follow-up study which recruited students from a variety of urban and rural, public and private high schools from Castelló. Ethical approval was obtained from University Ethics Committees; participants provided written informed consent [21, 22].

### Sociodemographic and disease-related parameters

The GRAS data collection contains comprehensive information regarding sociodemographic and disease-related parameters, acquired through detailed examination, semi-structured interviews, telephone consultations, questionnaires, and complete collection of hospitalization letters, allowing meticulous double-checking of patients’ self-reports [19, 20]. **Chlorpromazine equivalents** as indicator of present medication/disease severity and **past suicide attempts** as measure of severe self-aggression were employed for sample characterization and group comparison. **Premorbid intelligence** was estimated using *MWT-B (Mehrfachwahl-Wortschatz-Intelligenztest-B)*, and for **current cognitive symptoms**, a cognitive composite score was calculated, based on reasoning *(Leistungsprüfsystem-subtest-3)*, executive function *(Trail-Making-B)* and verbal learning and memory *(VLMT)* [18, 19].

### Environmental risk exposure

#### Schizophrenia subjects

Specific information was derived from history-taking and semi-structured interviews with patients and relatives/caretakers (GRAS-Manual) [19, 20] and from *SCID-I*. Each patient was dichotomously classified as having/not having been exposed premorbid and until age 18 years to **severe physical abuse** (comprising unpredictability of violence, injury due to physical reprimand or objects for corporal punishment), **sexual abuse** (forced touches, kissing, attempted or real rape), **migration** (subjects immigrating to Germany), **neurotrauma** (traumatic brain injury of all severity grades), **perinatal complications** (pregnancy, delivery, early postnatal life), **any cannabis consumption** and **alcohol abuse** [23]. To operationalize **urbanicity** until age 18, information on place of residence and relocation was collected from discharge letters, social history, telephone interviews/return mail (questionnaire). Total urbanicity score was dichotomously divided into rural vs. urban residence [18]. In case of contradictory or missing information, patients were excluded from respective analyses. Single risk factors with highest impact over all samples were accumulated to investigate combined influence on aggression.

#### General population subjects

**Physical** and **sexual abuse** was assessed by the shortened version of *Childhood Trauma Questionnaire (CTQ)* [24] and dichotomously recorded (never/any), as was **migration** (not born in Spain), **alcohol** (*Alcohol Use Disorders Identification Test - AUDIT* ≥ 4) [25] and any **cannabis consumption**. Data regarding perinatal complications, neurotrauma and urbanicity were unavailable.

### Measures of violent aggression and criminal conduct

#### Schizophrenia subjects

**Fig. 1:**
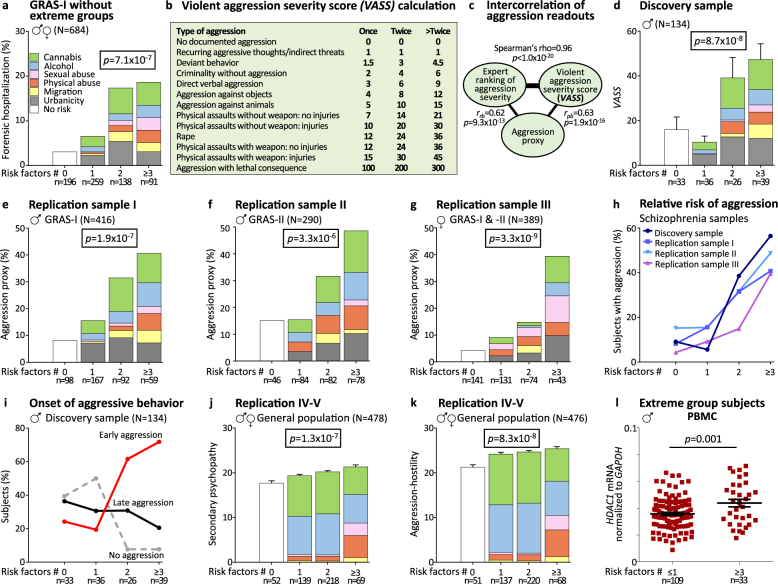
**Multiple environmental hits before adulthood predict violent aggression in mentally ill subjects as well as in the general population – Results from 6 independent samples**. **a** Distribution of forensic hospitalization in the discovery sample (see results) suggested a substantial impact of environmental risk accumulation on violent aggression, a finding replicated in the remaining GRAS sample (GRAS-I males and females minus extreme group subjects of the discovery sample). Note the ‘stair pattern’ upon stepwise increase in risk factors; stacked-charts illustrate risk factor composition in the respective groups (including all risk factors of each individual in the respective risk group). Each color represents a particular risk (same legend for **d**–**g** and **j**–**k**); *χ*^*2*^ test (two-sided). **b** Brief presentation of the violent aggression severity score, *VASS*, ranging from no documented aggression to lethal consequences of violent aggression with relative weight given to severity of aggression and number of registered re-occurrences. **c** Highly significant intercorrelation of violent aggression measures used in the present paper. **d** Application of *VASS* to risk accumulation in the discovery sample; Kruskal-Wallis*-H* test (two-sided). **e–g** Schizophrenia replication cohorts I–III: ‘stair pattern’ of aggression proxy in risk accumulation groups; all *χ*^*2*^ test (one-sided). **h** Comparative presentation of subjects (%) with violent aggression in risk accumulation groups across schizophrenia cohorts. **i** Comparative presentation of subjects (%) with violent aggression before (premorbid, ‘early’) or after schizophrenia onset (‘late’) vs. individuals without evidence of aggression (‘no’) in risk accumulation groups of the discovery sample. **j–k** General population replication cohorts IV and V: ‘stair pattern’ of aggression proxies, *LSRP* secondary psychopathy score (**j**) and aggression-hostility factor of *ZKPQ-50-CC* (**k**) in risk accumulation groups; Kruskal-Wallis-*H* test (one-sided). **l** *HDAC1* mRNA levels in PBMC of male extreme group subjects as available for analysis; Student’s *t* test (one-sided)

History of **forensic hospitalization** or **conviction** for battery, sexual assault, manslaughter, murder (at least once in life time) was used as violent aggression proxy. For cross-validation of this dichotomous variable, a continuous measure, the **violent aggression severity score** (*VASS*), based on questionnaires, interviews and charts, was generated and applied to the discovery sample. The *VASS* in turn was cross-validated by an intra-sample ranking of relative aggression severity by 2 independent raters (Fig. 1).

#### General population subjects

Secondary psychopathy of the *Levenson Self-Report Psychopathy Scale* (*LSRP*) [26], measuring antisocial aspects of psychopathy (rule-breaking; lack of effort towards socially rewarded behavior), and aggression-hostility factor of the *Zuckerman–Kuhlman Personality Questionnaire*, *shortened form (ZKPQ-50-CC)* [27], were used as proxies of violent aggression.

### Statistical analysis of environmental risk

Group differences for continuous variables were assessed using Mann-Whitney-*U* or Kruskal-Wallis-*H* test for comparison of > 2 groups. Frequency differences between groups were assessed using *χ*^*2*^-test or Fisher’s exact test. As trend tests, Jonckheere-Terpstra or Cochran-Armitage tests were applied. Covariates are explained in display items. Bonferroni correction accounted for multiple testing (*p* values withstanding correction denoted). Statistical analyses were performed using SPSS (v17.0; IBM-Deutschland GmbH, Munich, Germany), or R (v3.3.2; R-Foundation for Statistical Computing, Vienna, Austria).

### Methylation Array

Whole blood-derived DNA of extreme group individuals (*N* = 134) was analyzed by Infinium-HumanMethylation450K (Illumina Inc, CA, USA). Raw intensity data was preprocessed and SWAN (Subset-quantile Within Array Normalization) performed using Bioconductor package Minfi (v1.18.6) [28]. Probes with annotated single-nucleotide polymorphisms (SNPs) in CpG site or at single base extension sites were removed, leaving 467,971 probes total. To identify differentially methylated positions, a linear regression model using limma (v3.28.17) Bioconductor package [29] was fit. Covariates were age, medication and estimated cell proportions (monocytes, granulocytes, CD4T, CD8T, natural killer, and B-cells), calculated using Cell Counts Function in Minfi package [30]. A total of *N* = 129 individuals were finally included for the analyses since two samples were dropped based on separate clustering in principal component analysis and information regarding medication was not available for three samples. All analyses were performed in R.

### PBMC isolation and *HDAC1* assay

PBMC were isolated from morning blood, collected into CPDA-vials (Citrate-Phosphate-Dextrose-Adenine, Sarstedt, Germany), applying standard Ficoll-Paque-Plus isolation (GE-Healthcare, Munich, Germany). Total RNA extraction was done using miRNeasy Mini-kit (Qiagen, Hilden, Germany). For reverse transcription, 200ng RNA was applied using a mixture of oligo(dT)/hexamers, dNTPs, DTT and 200U SuperscriptIII (Life Technologies GmbH, Darmstadt, Germany). *HDAC1* expression was measured using quantitative real-time PCR. The cDNA was diluted 1:12.5 in 10 µl reaction-mix, containing 5 µl of SYBR-green (Life Technologies) and 1pmol/primer:

*HDAC1*-Fw: 5′-AAATTCTTGCGCTCCATCCG-3′

*HDAC1*-Rv: 5′-CAGGCCATCGAATACTGGACA-3′

*GAPDH*-Fw: 5′-CTGACTTCAACAGCGACACC-3′

*GAPDH*-Rv: 5′-TGCTGTAGCCAAATTCGTTGT-3′

Technical triplicates were run on LightCycler480 (Roche-Diagnostics GmbH, Mannheim, Germany). Relative *HDAC1* expression was calculated by the threshold-cycle method (LightCycler480 Software1.5.0SP3-Roche) and normalization to the housekeeping gene *GAPDH* was performed. After examination for outliers, Student’s *t* test was used to compare groups using Prism4 (GraphPad-Software; San Diego, CA, USA).

## Results

The environmental risk factors evaluated in this study comprise urbanicity, migration, perinatal complications, physical maltreatment, sexual abuse, traumatic brain injury, cannabis consumption and alcohol abuse. Contacting male extreme group subjects of GRAS (with low vs. high environmental risk before age 18; discovery sample; *N* = 134) [18] for a planned epigenetic follow-up, we found 27% of high-risk individuals in forensic units in contrast to only 6% of low-risk subjects (*p* < 0.001; *χ*^*2*^-test, two-sided). This finding was replicated in the remaining GRAS-I sample (GRAS-I males and females minus extreme group subjects), where a stepwise increase in lifetime prevalence of forensic hospitalization was seen upon risk accumulation (Fig. 1a).

This observation made us wonder whether we would find a strong intercorrelation between the here investigated environmental risks. To test for multicollinearity between the risk factors included in the accumulation models, we calculated the variance inflation factor (VIF) for each sample. Our results suggest that none of the included factors significantly collinears with any other (for each sample VIF ≤ 1.28), allowing us to include them in our models.

We hypothesized that forensic hospitalization reflects violent aggression. To quantify this trait, and in absence of established instruments for comprehensive retrospective analysis of violent aggression, we generated the *VASS* (Fig. 1b). Information for *VASS* was extracted for all discovery individuals (*N* = 134) from detailed history, available in the GRAS database [19, 20], and additional extensive chart study based on original medical documents over lifetime. *VASS* ranges from no documented aggression to lethal consequences of violent aggression. Relative weight is given to severity of aggression and number of registered re-occurrences. For first cross-validation of this new tool, an intra-sample expert ranking of relative aggression severity in the discovery sample was performed by 2 independent psychologists (unaware of environmental risk status of subjects under study), yielding Spearman’s rho = 0.97 for interrater reliability and rho = 0.96 for intercorrelation with *VASS* (Fig. 1c). Inspection of *VASS* values in the discovery sample upon risk accumulation again demonstrates the ‘stair pattern’ (Fig. 1d).

Since not all information was available as detailed for the schizophrenia replication samples of GRAS-I and -II as for the discovery sample, we introduced a dichotomous aggression proxy, including history of forensic hospitalization and/or conviction for battery, sexual assault, manslaughter or murder (at least once in lifetime). Intercorrelation with *VASS* and expert ranking, respectively, resulted in *r*_*pb*_ = 0.63 (point-biserial) and *r*_*rb*_ = 0.62 (rank-biserial) (Fig. 1c). Applying this proxy to replication samples I-III (GRAS-I males without discovery sample, GRAS-II males, GRAS-I&II females), consistently yielded the ‘stair pattern’ upon risk accumulation, even though at slightly lower level in females (Fig. 1e–g). The percentage of subjects with documented aggression increases with the number of risk factors, strikingly similar in all schizophrenia cohorts (Fig. 1h). Important for future preventive measures in *at-risk* subjects is the observation that a single risk factor (independent of its kind) is still compensated for (Fig. 1h). When comparing subjects with 0 vs. ≥3 environmental factors over all schizophrenia samples, the odds ratio for violent aggression (based on aggression proxy) amounts to 10.5. Details on sociodemographic and disease-related variables, as well as on the various highly intercorrelating measures of violent aggression in the environmental risk accumulation groups in discovery and replication samples are given in Tables 1 and 2. Whereas no consistent differences in premorbid intelligence, present cognition (cognitive composite), and chlorpromazine equivalents (relative amount of antipsychotics) emerge among groups, age tends to be lower and suicidality to occur more frequently with increasing pre-adult environmental risk exposure in the schizophrenia cohorts, which is not unexpected considering our previous report [18] (Table 1). A remarkable increase in all available measures of violent aggression becomes obvious upon accumulation of environmental risk (final model consisting of urbanicity, migration, physical and sexual abuse, alcohol and cannabis), reflected by highly significant *p* values in group and trend statistics throughout samples (Table 2).

**Table 1:** Presentation of environmental risk groups in discovery and replication samples: sociodemographic and disease-related measures

	No risk factors	1 risk factor	2 risk factors	≥3 risk factors	*p* value (*H*/χ^2^)
**Discovery sample**^**a**^ ***(N*** = ***121***–***134)***					
**Male schizophrenic subjects**	***n*** = ***30***–***33***	***n*** = ***32***–***36***	***n*** = ***24***–***26***	***n*** = ***35***–***39***	
Age (years)^b^	33.09 (10.24)	35.68 (11.23)	31.47 (8.27)	32.46 (8.66)	*p* = 0.630 (*H* = 1.73)
Premorbid intelligence *MWT-B*^c^	103.23 (16.57)	101.09 (11.80)	104.48 (14.36)	97.42 (14.91)	*p* = 0.172 (*H* = 5.00)
Cognitive composite score^d^	−0.05 (1.13)	−0.49 (1.07)	0.22 (0.72)	0.03 (1.00)	*p* = 0.651 (*H* = 1.64)
Chlorpromazine equivalents	751.09 (696.52)	771.87 (1227.51)	674.28 (508.49)	648.83 (569.38)	*p* = 0.769 (*H* = 1.13)
Suicidality^e^	11 (33.3%)	8 (23.5%)	9 (34.6%)	14 (36.8%)	*p* = 0.651 (χ^2^ = 1.64)
**Replication sample I*(N*** = ***392***–***411)***					
**GRAS I male schizophrenic subjects**	***n*** = ***91***–***98***	***n*** = ***156***–***166***	***n*** = ***91***–***92***	***n*** = ***53***–***59***	
Age (years)^b^	46.94 (12.26)	39.65 (12.50)	34.51 (10.18)	32.85 (8.38)	*p* = 1.6 x 10^−5^ (*H* = 24.87)
Premorbid intelligence *MWT-B*^c^	105.35 (17.09)	103.32 (15.87)	101.00 (14.26)	99.23 (15.10)	*p* = 0.085 (*H* = 6.61)
Cognitive composite score^d^	0.15 (1.12)	0.75 (1.01)	0.10 (0.93)	−0.01 (0.89)	*p* = 0.873 (*H* = 0.70)
Chlorpromazine equivalents	611.92 (571.29)	703.38 (585.70)	686.03 (608.01)	836.07 (622.14)	*p* = 0.059 (*H* = 7.43)
Suicidality^e^	23 (24.2%)	57 (34.5%)	33 (36.6%)	33 (55.9%)	*p* = 0.001 (χ^2^ = 16.11)
**Replication sample II** ***(N*** = ***238***–***290)***					
**GRAS II male schizophrenic subjects**	***n*** = ***36***–***46***	***n*** = ***68***–***84***	***n*** = ***67***–***82***	***n*** = ***67***–***78***	
Age (years)^b^	45.57 (15.02)	42.17 (13.83)	38.50 (14.08)	35.75 (10.52)	*p* = 0.011 (*H* = 11.20)
Premorbid intelligence *MWT-B*^c^	98.09 (14.46)	102.29 (16.03)	100.48 (13.36)	96.39 (9.29)	*p* = 0.184 (*H* = 4.84)
Cognitive composite score^d^	−0.23 (1.24)	−0.08 (1.03)	−0.01 (0.84)	−0.08 (0.96)	*p* = 0.816 (*H* = 0.94)
Chlorpromazine equivalents	629.15 (513.31)	747.35 (629.02)	689.31 (717.18)	713.84 (532.66)	*p* = 0.629 (*H* = 1.74)
Suicidality^e^	8 (20.5%)	12 (14.6%)	25 (31.6%)	25 (34.2%)	*p* = 0.018 (χ^2^ = 10.08)
**Replication sample III** (***N*** = ***345***–***386***)					
**GRAS I-II female schizophrenic subjects**	***n*** = ***125***–***140***	***n*** = ***118***–***130***	***n*** = ***65***–***71***	***n*** = ***37***–***43***	
Age (years)^b^	43.44 (11.75)	46.67 (12.90)	40.84 (12.71)	36.53 (11.31)	*p* = 0.003 (*H* = 13.83)
Premorbid intelligence *MWT-B*^c^	103.53 (14.19)	104.04 (14.28)	102.96 (15.84)	99.10 (15.41)	*p* = 0.147 (*H* = 5.37)
Cognitive composite score^d^	0.03 (0.96)	0.09 (0.99)	0.24 (0.99)	−0.21 (1.00)	*p* = 0.164 (*H* = 5.11)
Chlorpromazine equivalents	536.52 (579.61)	564.04 (506.21)	620.48 (628.30)	650.87 (477.23)	*p* = 0.167 (*H* = 5.07)
Suicidality^e^	45 (33.6%)	59 (46.5%)	33 (46.5%)	22 (53.7%)	*p* = 0.052 (χ^2^ = 7.72)
**Replication sample IV** ***(N*** = ***299)***					
**General population**	***n*** = ***39***	***n*** = ***83***	***n*** = ***133***	***n*** = ***44***	
Age (years)	26.44 (4.81)	25.93 (2.46)	25.56 (3.50)	25.25 (3.64)	*p* = 0.117 (*H* = 5.89)
Gender, female/male (% male)	29/10 (25.6%)	51/32 (38.6%)	78/55 (41.4%)	18/26 (59.1%)	
**Replication sample V** ***(N*** = ***177***–***183***)					
**General population**	***n*** = ***13***	***n*** = ***54***–***56***	***n*** = ***86***–***89***	***n*** = ***24***–***25***	
Age (years)	20.54 (0.88)	20.63 (0.98)	20.85 (1.12)	20.88 (1.15)	*p* = 0.696 (*H* = 1.44)
Gender, female/male (% male)	7/6 (46.2%)	41/15 (26.8%)	56/33 (37.1%)	20/5 (20.0%)	

Data are uncorrected means (SD) or n (%); for statistical analysis, Kruskal-Wallis-*H*, χ^*2*^, or Fisher’s exact test was used, all *p* values two-sided; Bonferroni-corrected *p* values <0.01 are considered significant and underlined; because of missing data, sample sizes vary;

^a^note regarding discovery sample: extreme groups of our previous study [18] differ slightly due to elimination of birth complications and neurotrauma, but inclusion of alcohol in the present study;

^b^corrected for age at disease onset;

^c^ *MWT-B=Mehrfachwahl-Wortschatz-Intelligenztest-B;*

^d^cognitive composite score consists of reasoning *(Leistungsprüfsystem-subtest-3)*, executive function *(Trail-Making Test B)*, verbal learning & memory test (*VLMT*) [18]; corrected for age, *PANSS* negative score, and chlorpromazine equivalents (standardized residuals after linear regression);

^e^suicidality=individuals with past suicide attempts

**Table 2:** Effect of environmental risk factor accumulation on measures of aggressive behavior in schizophrenic and general population subjects

	No risk factors	1 risk factor	2 risk factors	≥3 risk factors	*p* value (χ^2^/*H*)	*p* value (χ^2^/*J*)^a^
**Discovery sample**^**b**^ ***(N*** = ***134)***						
**Male schizophrenic subjects**	***n*** = ***33***	***n*** = ***36***	***n*** = ***26***	***n*** = ***39***		
History of forensic hospitalization	1 (3.0%)	1 (2.8%)	6 (23.1%)	14 (35.9%)	*p* = 5.4 x 10^−5†^ (χ^2^ = 20.82)	*p* = 1.5 x 10^−5^ (χ^2^ = 18.71)
Aggression proxy^c^	3 (9.1%)	2 (5.6%)	10 (38.5%)	22 (56.4%)	*p* = 4.9 x 10^−7^ (χ^2^ = 32.14)	*p* = 1.3 x 10^−7^ (χ^2^ = 27.80)
Violent aggression severity score *(VASS)*	16.03 (32.17)	10.49 (15.50)	39.21 (45.38)	47.45 (43.14)	*p* = 8.7 x 10^−8^ (*H* = 35.70)	*p* = 9.2 x 10^−8^ (*J* = 4671.5)
Aggression before schizophrenia^d^	8 (24.2%)	7 (19.4%)	16 (61.5%)	28 (71.8%)	*p* = 1.8 x 10^−6^ (χ^2^ = 29.51)	*p* = 7.4 x 10^−7^ (χ^2^ = 24.52)
**Replication sample I** (***N*** = ***411***–***416***)						
**GRAS I male schizophrenic subjects**	***n*** = ***96***–***98***	***n*** = ***165***–***167***	***n*** = ***91***–***92***	***n*** = ***59***		
History of forensic hospitalization	5 (5.2%)	14 (8.5%)	21 (23.1%)	12 (20.3%)	*p* = 0.0001 (χ^2^ = 19.51)	*p* = 4.7 x 10^−5^ (χ^2^ = 15.25)
Aggression proxy^c^	8 (8.2%)	26 (15.6%)	29 (31.5%)	24 (40.7%)	*p* = 1.9 x 10^−7^ (χ^2^ = 32.71)	*p* = 8.9 x 10^−9^ (χ^2^ = 31.73)
**Replication sample II** (***N*** = ***289***–***290***)						
**GRAS II male schizophrenic subjects**	***n*** = ***46***	***n*** = ***84***	***n*** = ***81***–***82***	***n*** = ***78***		
History of forensic hospitalization	5 (10.9%)	10 (11.9%)	26 (32.1%)	32 (41.0%)	*p* = 6.8 x 10^−6^ (χ^2^ = 25.26)	*p* = 8.6 x 10^−7^ (χ^2^ = 22.88)
Aggression proxy^c^	7 (15.2%)	13 (15.5%)	26 (31.7%)	38 (48.7%)	*p* = 3.3 x 10^−6^ (χ^2^ = 26.74)	*p* = 4.6 x 10^−7^ (χ^2^ = 24.10)
**Replication sample III** (***N*** = ***389***–***392***)						
**GRAS I–II female schizophrenic subjects**	***n*** = ***141***–***142***	***n*** = ***131***–***133***	***n*** = ***74***	***n*** = ***43***		
History of forensic hospitalization	1 (0.7%)	4 (3.0%)	4 (5.4%)	6 (14.0%)	*p* = 0.002^†^ (χ^2^ = 16.49)	*p* = 7.5 x 10^−5^ (χ^2^ = 14.37)
Aggression proxy^c^	6 (4.3%)	12 (9.2%)	11 (14.9%)	17 (39.5%)	*p* = 3.3 x 10^−9^ (χ^2^ = 40.96)	*p* = 3.5 x 10^−9^ (χ^2^ = 33.56)
**Replication sample IV** (***N*** = ***293***–***295***)						
**General population**	***n*** = ***38***–***39***	***n*** = ***81***–***83***	***n*** = ***129***–***131***	***n*** = ***43***–***44***		
Secondary psychopathy score, *LSRP*^*e*^	17.46 (3.25)	19.12 (3.52)	20.17 (3.49)	20.57 (3.18)	*p* = 4.6 x 10^−5^ (*H* = 21.94)	*p* = 1.1 x 10^−5^ (*J* = 18371)
Aggression-hostility score, *ZKPQ*^*f*^	21.18 (3.68)	24.05 (3.27)	24.51 (4.07)	24.91 (3.62)	*p* = 3.4 x 10^−5^ (*H* = 21.28)	*p* = 4.5 x 10^−4^ (*J* = 17550.5)
**Replication sample V** (***N*** = ***183)***						
**General population**	***n*** = ***13***	***n*** = ***56***	***n*** = ***89***	***n*** = ***25***		
Secondary psychopathy score, *LSRP*^*e*^	18.54 (3.89)	19.73 (3.63)	20.38 (3.17)	22.76 (3.33)	*p* = 0.0009 (*H* = 15.01)	*p* = 0.0003 (*J* = 6721)
Aggression-hostility score, *ZKPQ*^*f*^	21.62 (3.10)	24.50 (4.95)	25 (4.23)	26.32 (3.41)	*p* = 0.004 (*H* = 11.61)	*p* = 0.003 (*J* = 6438)
**General population, replication samples IV & V together**						
**General population, males**	***n*** = ***16***	***n*** = ***46***–***47***	***n*** = ***86***–***88***	***n*** = ***31***		
Secondary psychopathy score, *LSRP*^*e*^	17.25 (4.46)	19.57 (3.16)	20.42 (3.31)	20.39 (2.93)	*p* = 0.022 (*H* = 8.06)	*p* = 0.011 (*J* = 6253.5)
Aggression-hostility score, *ZKPQ*^*f*^	20.38 (2.94)	23.63 (3.30)	25.09 (4.16)	24.90 (3.77)	*p* = 3.29 x 10^−5^ (*H* = 21.98)	*p* = 1.02 x 10^−4^ (*J* = 6821)
**General population, females**	***n*** = ***35***–***36***	***n*** = ***91***–***92***	***n*** = ***132***	***n*** = ***37***–***38***		
Secondary psychopathy score, *LSRP*^*e*^	17.94 (2.88)	19.26 (3.76)	20.15 (3.40)	22.16 (3.55)	*p* = 8.35 x 10^−7^ (*H* = 29.60)	*p* = 9.6 x 10^−8^ (*J* = 19223.5)
Aggression-hostility score, *ZKPQ*^*f*^	21.71 (3.71)	24.54 (4.34)	24.45 (4.11)	25.86 (3.42)	*p* = 1.20 x 10^−4^ (*H* = 19.28)	*p* = 0.0013 (*J* = 17067)

Urbanicity, migration, physical abuse, sexual abuse, problematic alcohol use, and cannabis use are included in the accumulation model; data are uncorrected means (SD) or n (%); for statistical analysis, Kruskal-Wallis-*H*, χ^*2*^, or Fisher’s exact test was used; *p* values <0.01 are considered significant and underlined; because of missing data, sample sizes vary;

^a^to test for statistical trends, the Cochran-Armitage trend (qualitative traits) or Jonckheere-Terpstra trend (quantitative traits) test was used; for replication samples, testing was one-sided;

^b^note regarding discovery sample: extreme groups of our previous study [18] differ slightly due to elimination of birth complications and neurotrauma, but inclusion of alcohol in the present study;

^c^any conviction for battery, sexual assault, manslaughter, murder, or a history of forensic hospitalization;

^d^deviant behavior, criminality, verbal, physical, or sexual aggression, at least half a year (mean=13.69 years, SD=10.10) before first psychotic episode;

^e^*LSRP=Levenson Self-Report Psychopathy Scale*; ^f^*ZKPQ-50-CC=Zuckerman-Kuhlman Personality Questionnaire*;

^†^two-sided Fisher’s exact test for group size estimations <5.

For analyzing onset of aggressive behavior, the extensive information on aggression available in the discovery sample was exploited. Early aggression (any aggression documented before age 18 years and well before schizophrenia onset) clearly increased upon ≥2 risk factors, whereas aggression seen only later in life seemed independent of early environmental risk (Fig. 1i). Therefore, we hypothesized that violent aggression upon risk accumulation may be unrelated to mental disease.

To test this hypothesis, we had the chance to analyze 2 well-characterized independent samples (replication IV and V; Tables 1 and 2) of young individuals from the Spanish general population. Since data on criminal conduct could not be obtained in these cohorts, we had to use alternative, psychometrically validated instruments as aggression proxies, namely *LSRP* secondary psychopathy score [26], measuring rule-breaking and lack of effort towards socially rewarded behavior, and the aggression-hostility factor of *ZKPQ-50-CC* [27]. Urbanicity as risk factor was unavailable in these samples (reducing the model to 5 of the 6 risk factors explored in schizophrenia, that is migration, physical maltreatment, sexual abuse, alcohol and cannabis). We also note that subjects were younger and as academics probably higher educated as compared to the disease cohorts. Despite these mitigating facts, and despite employing individuals of another country, the expected ‘stair pattern’ still emerged clearly for both proxies, likely suggesting generalizability of these findings (Fig. 1j,k; Tables 1 and 2). Data given here for the general population samples (replications IV and V) are based on both males and females. In addition, evaluating men and women separately (taking both general population cohorts together) yielded significant results for both genders (Table 2 bottom).

Addressing the composition of risk factors among groups across cohorts, we obtained a comparable pattern throughout schizophrenia samples (stacked-charts; Fig. 1a,d-g). In the general population subjects, particularly alcohol and cannabis consumption (classified as ‘secondary hits’) predominated (Fig. 1j,k) which also seem to play an appreciable role in schizophrenia cohorts. Therefore, we wondered whether separate analysis of risk accumulation, integrating only primary vs. only secondary hits, would still result in significant effects on aggression. For all schizophrenia samples individually, group difference and trend remained highly significant (not shown). Taking all schizophrenia subjects together (*N* > 1200), the aggression proxy yields for the accumulation model, built on primary risks only (urbanicity, migration, physical and sexual abuse), *p* = 4.5 × 10^−17^ (*χ*^*2*^ = 75.28) and *p < *2.2 × 10^−16^ (*χ*^*2*^ = 68.28), for group differences and trend, respectively. The corresponding results for secondary risk factors (alcohol, cannabis) in schizophrenia are *p* = 6.6 × 10^−19^ (*χ*^*2*^ = 83.71) and *p* < 2.2 × 10^−16^ (*χ*^*2*^ = 83.40). Analogously, taking all general population subjects together (*N* > 530), we obtain for *LSRP* with primary risks (urbanicity not available) *p* = 0.002 (*H* = 12.65) and *p* = 0.0003 (*J* = 33774.5), and with secondary risks *p* = 1.3 × 10^−4^ (*H* = 17.92) and *p* = 5.3 × 10^−5^ (*J* = 42412.5) for group differences and trend. Also here, significance was already reached with separate analysis of both cohorts (not shown).

For deciding on the accumulation model, we had initially screened all individual risk factors of our ‘primary plus secondary risk factor model’ separately in both schizophrenia and general population cohorts to get an estimation of their relative impact (Tables 3a,3b,3c). Perinatal complications and neurotrauma before the age of 18 years were unavailable for general population subjects. Since these risks showed the lowest overall impact on aggression proxies in schizophrenia, we decided not to include them in our present accumulation model.

**Table 3a:** Effect of single environmental risk factors on measures of aggressive behavior in schizophrenic and general population subjects

	Perinatal hit		*p* value (χ^2^/*Z*)	Urbanicity		*p* value (χ^2^/*Z)*	Migration		*p* value (χ^2^/*Z)*
	No	Yes		Rural	Urban		No	Yes	
**Discovery sample** ***(N*** = ***134)***									
**Male schizophrenic subjects**	***n*** = ***77***	***n*** = ***57***		***n*** = ***66***	***n*** = ***68***		***n*** = ***114***	***n*** = ***20***	
History of forensic hospitalization	12 (15.6%)	10 (17.5%)	*p* = 0.762 (χ^2^ = 0.09)	7 (10.6%)	15 (22.1%)	*p* = 0.074 (χ^2^ = 3.20)	12 (10.5%)	10 (50.0%)	*p* = 0.0001^†^ (χ^2^ = 19.32)
Aggression proxy^a^	18 (23.4%)	19 (33.3%)	*p* = 0.202 (χ^2^ = 1.63)	11 (16.7%)	26 (38.2%)	*p* = 0.005 (χ^2^ = 7.80)	21 (18.4%)	16 (80.0%)	*p* = 1.3 x 10^−8^ (χ^2^ = 32.28)
Violent aggression severity score (*VASS*)	23.01 (37.43)	35.17 (39.23)	*p* = 0.003 (*Z* = −2.96)	22.86 (38.29)	33.35 (38.35)	*p* = 0.019 (*Z* = −2.35)	23.16 (33.73)	56.83 (51.12)	*p* = 0.0005 (*Z* = −3.46)
Aggression before schizophrenia^b^	25 (32.5%)	34 (59.6%)	*p* = 0.002 (χ^2^ = 9.82)	24 (36.4%)	35 (51.5%)	*p* = 0.078 (χ^2^ = 3.10)	46 (40.4%)	13 (65.0%)	*p* = 0.041 (χ^2^ = 4.20)
**Replication sample I** ***(N*** = ***438***–***606)***									
**GRAS I male schizophrenic subjects**	***n*** = ***374***–***381***	***n*** = ***222***–***223***		***n*** = ***262***–***266***	***n*** = ***176***–***178***		***n*** = ***542***–***550***	***n*** = ***56***	
History of forensic hospitalization	51 (13.6%)	25 (11.3%)	*p* = 0.201 (χ^2^ = 0.71)	28 (10.7%)	25 (14.2%)	*p* = 0.134 (χ^2^ = 1.23)	59 (10.9%)	17 (30.4%)	*p* = 1.6 x 10^−5^ (χ^2^ = 17.35)
Aggression proxy^a^	81 (21.3%)	48 (21.5%)	*p* = 0.470 (χ^2^ < 0.01)	43 (16.2%)	45 (25.3%)	*p* = 0.009 (χ^*2*^ = 5.58)	102 (18.5%)	27 (48.2%)	*p* = 1.2 x 10^−7^ (χ^*2*^ = 26.70)
**Replication sample II** ***(N*** = ***316***–***320)***									
**GRAS II male schizophrenic subjects**	***n*** = ***219***–***220***	***n*** = ***99***		***n*** = ***182***–***183***	***n*** = ***134***		***n*** = ***282***–***283***	***n*** = ***37***	
History of forensic hospitalization	55 (25.1%)	25 (25.3%)	*p* = 0.490 (χ^*2*^ < 0.01)	41 (22.5%)	39 (29.1%)	*p* = 0.092 (χ^*2*^ = 1.77)	65 (23.0%)	15 (40.5%)	*p* = 0.011 (χ^*2*^ = 5.33)
Aggression proxy^a^	64 (29.1%)	28 (28.3%)	*p* = 0.442 (χ^*2*^ = 0.02)	45 (24.6%)	47 (35.1%)	*p* = 0.021 (χ^*2*^ = 4.13)	76 (26.9%)	16 (43.2%)	*p* = 0.019 (χ^*2*^ = 4.29)
**Replication sample III** ***(N*** = ***424***–***503)***									
**GRAS I–II female schizophrenic subjects**	***n*** = ***269***–***300***	***n*** = ***200***		***n*** = ***267***–***268***	***n*** = ***157***–***159***		***n*** = ***450***–***454***	***n*** = ***49***	
History of forensic hospitalization	11 (3.7%)	5 (2.5%)	*p* = 0.234 (χ^*2*^ = 0.53)	6 (2.2%)	7 (4.4%)	*p* = 0.166^†^ (χ^*2*^ = 1.58)	14 (3.1%)	2 (4.1%)	*p* = 0.474^†^ (χ^*2*^ = 0.14)
Aggression proxy^a^	33 (11.1%)	23 (11.5%)	*p* = 0.452 (χ^*2*^ = 0.02)	25 (9.4%)	20 (12.7%)	*p* = 0.138 (χ^*2*^ = 1.19)	48 (10.7%)	8 (16.3%)	*p* = 0.117 (χ^*2*^ = 1.42)
**Replication sample IV–V** ***(N*** = ***513***–***551)***									
**General population**	**NA**	**NA**		**NA**	**NA**		***n*** = ***517***–***521***	***n*** = ***25***–***26***	
Secondary psychopathy score – *LSRP*^*c*^	NA	NA		NA	NA		19.79 (3.53)	20.96 (3.30)	*p* = 0.047 (*Z* = −1.68)
Aggression-hostility score – *ZKPQ*^*d*^	NA	NA		NA	NA		24.25 (4.04)	24.81 (4.55)	*p* = 0.344 (*Z* = −0.40)

Data are uncorrected means (SD) or n (%); for statistical analysis, Mann-Whitney-*U*, χ^*2*^, or Fisher’s exact test was used; significant *p* values are underlined; for replication samples, testing was one-sided; because of missing data, sample sizes vary;

^a^any conviction for battery, sexual assault, manslaughter and murder, or a history of forensic hospitalization;

^b^deviant behavior, criminality, verbal, physical, or sexual aggression at least half a year (mean=13.69 years, SD=10.10) before first psychotic episode;

^c^*LSRP=Levenson Self-Report Psychopathy Scale*;

^d^*ZKPQ-50-CC=Zuckerman-Kuhlman Personality Questionnaire*;

^†^Fisher’s exact test upon group size estimations <5; NA=information not available.

**Table 3b:** Effect of single environmental risk factors on measures of aggressive behavior in schizophrenic and general population subjects (continued)

	Neurotrauma		*p* value (χ^*2*^/*Z*)	Physical abuse		*p* value (χ^*2*^/*Z*)	Sexual abuse		*p* value (χ^2^/*Z*)
	No	Yes		No	Yes		No	Yes	
**Discovery sample** ***(N*** = ***134)***									
**Male schizophrenic subjects**	***n*** = ***66***	***n*** = ***68***		***n*** = ***112***	***n*** = ***22***		***n*** = ***123***	***n*** = ***11***	
History of forensic hospitalization	5 (7.6%)	17 (25.0%)	*p* = 0.006 (χ^*2*^ = 7.41)	16 (14.3%)	6 (27.3%)	*p* = 0.203^†^ (χ^*2*^ = 2.26)	19 (15.4%)	3 (27.3%)	*p* = 0.388^†^ (χ^*2*^ = 1.03)
Aggression proxy^a^	10 (15.2%)	27 (39.7%)	*p* = 0.001 (χ^2^ = 10.10)	28 (25.0%)	9 (40.9%)	*p* = 0.127 (χ^2^ = 2.33)	32 (26.0%)	5 (45.5%)	*p* = 0.176^†^ (χ^2^ = 1.91)
Violent aggression severity score (*VASS*)	16.42 (27.03)	39.60 (44.39)	*p* = 4.5 x 10^−5^ (*Z* = −4.08)	24.71 (36.73)	45.89 (43.35)	*p* = 0.003 (*Z* = −2.96)	26.19 (35.98)	50.50 (57.95)	*p* = 0.071 (*Z* = −1.80)
Aggression before schizophrenia^b^	16 (24.2%)	43 (63.2%)	*p* = 5.5 x 10^−6^ (χ^*2*^ = 20.66)	45 (40.2%)	14 (63.6%)	*p* = 0.043 (χ^*2*^ = 4.11)	51 (41.5%)	8 (72.7%)	*p* = 0.059^†^ (χ^*2*^ = 4.01)
**Replication sample I** ***(N*** = ***567***–***606)***									
**GRAS I male schizophrenic subjects**	***n*** = ***263***–***265***	***n*** = ***304***–***307***		***n*** = ***535***–***543***	***n*** = ***63***		***n*** = ***547***–***555***	***n*** = ***38***	
History of forensic hospitalization	40 (15.2%)	31 (10.2%)	*p* = 0.036 (χ^*2*^ = 3.52)	60 (11.2%)	16 (25.4%)	*p* = 0.0007 (χ^*2*^ = 10.22)	67 (12.2%)	8 (21.1%)	*p* = 0.098^†^ (χ^*2*^ = 2.46)
Aggression proxy^a^	59 (22.3%)	60 (19.5%)	*p* = 0.212 (χ^*2*^ = 0.64)	107 (19.7%)	22 (34.9%)	*p* = 0.003 (χ^*2*^ = 7.80)	118 (21.3%)	10 (26.3%)	*p* = 0.232 (χ^*2*^ = 0.54)
**Replication sample II** ***(N*** = ***293***–***320)***									
**GRAS II male schizophrenic subjects**	***n*** = ***144***	***n*** = ***175***–***176***		***n*** = ***216***–***217***	***n*** = ***102***		***n*** = ***271***–***272***	***n*** = ***22***	
History of forensic hospitalization	36 (25.0%)	44 (25.1%)	*p* = 0.489 (χ^*2*^ < 0.01)	45 (20.8%)	34 (33.3%)	*p* = 0.008 (χ^*2*^ = 5.80)	66 (24.4%)	7 (31.8%)	*p* = 0.223 (χ^*2*^ = 0.61)
Aggression proxy^a^	42 (29.2%)	50 (28.4%)	*p* = 0.441 (χ^*2*^ = 0.02)	51 (23.5%)	40 (39.2%)	*p* = 0.002 (χ^*2*^ = 8.40)	76 (27.9%)	8 (36.4%)	*p* = 0.200 (χ^*2*^ = 0.71)
**Replication sample III** ***(N*** = ***494***–***502)***									
**GRAS I–II female schizophrenic subjects**	***n*** = ***319***–***322***	***n*** = ***177***–***178***		***n*** = ***433***–***437***	***n*** = ***65***		***n*** = ***397***–***400***	***n*** = ***97***–***98***	
History of forensic hospitalization	11 (3.4%)	5 (2.8%)	*p* = 0.356 (χ^*2*^ = 0.14)	10 (2.3%)	6 (9.2%)	*p* = 0.011^†^ (χ^*2*^ = 8.84)	10 (2.5%)	6 (6.1%)	*p* = 0.073^†^ (χ^*2*^ = 3.32)
Aggression proxy^a^	32 (10.0%)	24 (13.6%)	*p* = 0.117 (χ^*2*^ = 1.42)	41 (9.5%)	15 (23.1%)	*p* = 0.0006 (χ^*2*^ = 10.49)	34 (8.6%)	22 (22.7%)	*p* = 4.2 x 10^−5^ (χ^*2*^ = 15.46)
**Replication sample IV–V** ***(N*** = ***513***–***551)***									
**General population**	**NA**	**NA**		***n*** = ***453***–***456***	***n*** = ***95***–***96***		***n*** = ***505***	***n*** = ***42***–***44***	
Secondary psychopathy score – *LSRP*^*c*^	NA	NA		19.65 (3.49)	20.68 (3.37)	*p* = 0.004 (*Z* = −2.72)	19.72 (3.44)	21.07 (3.90)	*p* = 0.013 (*Z* = −2.23)
Aggression-hostility score – *ZKPQ*^*d*^	NA	NA		24.08 (4.01)	25.07 (4.23)	*p* = 0.023 (*Z* = −1.99)	24.19 (4.12)	24.83 (3.30)	*p* = 0.128 (*Z* = −1.13)

Data are uncorrected means (SD) or n (%); for statistical analysis, Mann-Whitney-*U*, χ^*2*^, or Fisher’s exact test was used; significant *p* values are underlined; for replication samples, testing was one-sided; because of missing data, sample sizes vary;

^a^any conviction for battery, sexual assault, manslaughter and murder, or a history of forensic hospitalization;

^b^deviant behavior, criminality, verbal, physical, or sexual aggression at least half a year (mean=13.69 years, SD=10.10) before first psychotic episode;

^c^*LSRP=Levenson Self-Report Psychopathy Scale*;

^d^*ZKPQ-50-CC=Zuckerman-Kuhlman Personality Questionnaire*;

^†^Fisher’s exact test upon group size estimations <5; NA=information not available

**Table 3c:** Effect of single environmental risk factors on measures of aggressive behavior in schizophrenic and general population subjects (continued)

	Problematic alcohol use		*p* value (χ^*2*^/*Z*)	Cannabis use		*p* value (χ^*2*^/*Z*)
	No	Yes		No	Yes	
**Discovery sample** ***(N*** = ***134)***						
**Male schizophrenic subjects**	***n*** = ***102***	***n*** = ***32***		***n*** = ***66***	***n*** = ***68***	
History of forensic hospitalization	14 (13.7%)	8 (25.0%)	*p* = 0.133 (χ^*2*^ = 2.26)	4 (6.1%)	18 (26.5%)	*p* = 0.001 (χ^*2*^ = 10.17)
Aggression proxy^a^	25 (24.5%)	12 (37.5%)	*p* = 0.152 (χ^*2*^ = 2.06)	9 (13.6%)	28 (41.2%)	*p* = 0.0004 (χ^*2*^ = 12.71)
Violent aggression severity score (*VASS*)	25.60 (38.59)	36.41 (37.79)	*p* = 0.022 (*Z* = −2.30)	16.30 (27.82)	39.72 (43.85)	*p* = 1.1 x 10^−5^ (*Z* = −4.39)
Aggression before schizophrenia^b^	37 (36.3%)	22 (68.8%)	*p* = 0.001 (χ^*2*^ = 10.42)	18 (27.3%)	41 (60.3%)	*p* = 0.0001 (χ^*2*^ = 14.82)
**Replication sample I** ***(N*** = ***575***–***582)***						
**GRAS I male schizophrenic subjects**	***n*** = ***472***–***477***	***n*** = ***103***–***104***		***n*** = ***319***–***323***	***n*** = ***257***–***259***	
History of forensic hospitalization	60 (12.7%)	15 (14.6%)	*p* = 0.307 (χ^*2*^ = 0.26)	28 (8.8%)	42 (16.3%)	*p* = 0.003 (χ^*2*^ = 7.63)
Aggression proxy^a^	91 (19.1%)	35 (33.7%)	*p* = 0.0005 (χ^*2*^ = 10.68)	48 (14.9%)	73 (28.2%)	*p* = 4.1 x 10^−5^ (χ^*2*^ = 15.50)
**Replication sample II** ***(N*** = ***293***–***294)***						
**GRAS II male schizophrenic subjects**	***n*** = ***204***	***n*** = ***89***–***90***		***n*** = ***146***	***n*** = ***147***–***148***	
History of forensic hospitalization	39 (19.1%)	35 (39.3%)	*p* = 0.0001 (χ^2^ = 13.41)	23 (15.8%)	51 (34.7%)	*p* = 9.5 x 10^−5^ (χ^*2*^ = 13.92)
Aggression proxy^a^	48 (23.5%)	38 (42.2%)	*p* = 0.0006 (χ^*2*^ = 10.54)	29 (19.9%)	57 (38.5%)	*p* = 0.0002 (χ^*2*^ = 12.35)
**Replication sample III** ***(N*** = ***466***–***490)***						
**GRAS I–II female schizophrenic subjects**	***n*** = ***436***–***440***	***n*** = ***30***		***n*** = ***401***–***405***	***n*** = ***85***	
History of forensic hospitalization	12 (2.7%)	3 (10.0%)	*p* = 0.063^†^ (χ^*2*^ = 4.81)	9 (2.2%)	7 (8.2%)	*p* = 0.011^†^ (χ^*2*^ = 8.04)
Aggression proxy^a^	40 (9.2%)	11 (36.7%)	*p* = 0.0001^†^ (χ^*2*^ = 21.77)	32 (8.0%)	23 (27.1%)	*p* = 2.3 x 10^−7^ (χ^*2*^ = 25.44)
**Replication sample IV–V** ***(N*** = ***513***–***551)***						
**General population**	***n*** = ***163***–***165***	***n*** = ***361***–***363***		***n*** = ***156***–***158***	***n*** = ***357***	
Secondary psychopathy score – *LSRP*^*c*^	18.89 (3.56)	20.37 (3.37)	*p* = 7.9 x 10^−6^ (*Z* = −4.32)	19.27 (3.59)	20.04 (3.48)	*p* = 0.013 (*Z* = −2.24)
Aggression-hostility score – *ZKPQ*^*d*^	23.15 (3.91)	24.90 (4.06)	*p* = 1.1 x 10^−6^ (*Z* = −4.73)	23.31 (4.47)	24.64 (3.78)	*p* = 0.0005 (*Z* = −3.47)

Data are uncorrected means (SD) or n (%); for statistical analysis, Mann-Whitney-*U*, χ^*2*^, or Fisher’s exact test was used; significant *p* values are underlined; for replication samples, testing was one-sided; because of missing data, sample sizes vary;

^a^any conviction for battery, sexual assault, manslaughter and murder or a history of forensic hospitalization;

^b^deviant behavior, criminality, verbal, physical, or sexual aggression at least half a year (mean=13.69 years, SD=10.10) before first psychotic episode;

^c^*LSRP=Levenson Self-Report Psychopathy Scale*;

^d^*ZKPQ-50-CC=Zuckerman-Kuhlman Personality Questionnaire*;

^†^Fisher’s exact test upon group size estimations <5; NA=information not available

Finally, we performed an epigenome-wide association scan to detect differential methylation of blood-derived DNA of selected extreme group individuals (discovery sample; *N* = 134; Fig. 1a), originally planned as epigenetic follow-up study [18]. This scan turned out to be negative. In fact, contrasting subjects either with high vs. low number of environmental hits or according to *VASS* median split yielded a single methylation difference upon lowering the Bonferroni threshold to 10^−6^ (Table 4). Similarly, when looking in an exploratory fashion (small/unbalanced group sizes) at individual risk factors separately, results were essentially negative (Table 4). Hits associated with migration were likely related to ethnicity rather than environmental risk, as reported recently [31]. The power of our sample size - even though in the range of suggestions [32] and despite extreme group comparison - may not have been sufficient to detect differences, also due to a vast underlying heterogeneity of individual methylation sites. Even the search for methylation differences of aggression-related candidate genes [33–35] turned out negative (not shown), putting the relative weight of phenotypical consequences (here violent aggression) vs. common methylation results in humans into perspective. In contrast, determining *HDAC1* mRNA levels in PBMC available from male extreme group subjects (≤1 vs. ≥3 risks) revealed a highly significant difference (*p* = 0.001), with higher levels in the high-risk (*N* = 33) compared to the low-risk group (*N* = 109) (Fig. 1l). This transcript encodes an enzyme of the histone deacetylase complex which serves as an overarching regulator of epigenetic processes. Indeed, peripheral *HDAC1* mRNA levels seem to be a more robust readout of epigenetic modifications in small sample sizes [36] as compared to specific methylation sites in the epigenome-wide association scan, and suggest lasting epigenetic alterations.

**Table 4:** Comparison of methylation data from whole blood-derived DNA of selected extreme group individuals (schizophrenia discovery sample; *N* = 134)

			Number of significant CpG sites^a^		
Factor		*n*	*p*<10^−8^	*p*<10^−7^	*p*<10^−6^
High vs. low # of environmental hits	High	64	0	0	0
	Low	65			
*VASS*	High	64	0	0	1
(Median split)	Low	65			cg23980294^b^
Perinatal hit	Yes	55	0	0	0
	No	74			
Urbanicity	Yes	63	0	0	1
	No	66			cg08446900^c^
Migration	Yes	19	5	+12	+73^d^
	No	110	cg19078576, cg24719005, cg06809544, cg25146017, cg17275700	cg15916004, cg17714025, cg14326196, cg11236526, cg15858239, cg18952796, cg12969644, cg13895765, cg12204732, cg12892004, cg19927816, cg05641882	cg07303244, cg18156204, cg13181928, cg04061117, cg13944175, cg24366557, cg10530883, cg17046577, cg16318053, cg04529370, cg08146323, cg15989068, cg09017434, cg06622999, cg09072859, cg02106850, cg14576062, cg10383019, cg22721334, cg15953602, cg14594187, cg06248560, cg23291534, cg10387551, cg05756220, cg14603345, cg25495650, cg06659727, cg20019985, cg20937139, cg09938511, cg12818557, cg09469566, cg14155416, cg17504999, cg15543566, cg13790603, cg17630392, cg00421139, cg04842426, cg26133769, cg16668359, cg03606215, cg20705321, cg00688962, cg05839235, cg02355420, cg22620221, cg10603275, cg13904970, cg19806642, cg08111167, cg07535928, cg18778433, cg14051544, cg01734112, cg18249173, cg18395636, cg13407975, cg05191076, cg11429292, cg18932726, cg15310492, cg23130097, cg09858188, cg15439862, cg13805537, cg25556464, cg13781843, cg04950301, cg12338417, cg14875171, cg08431899
Neurotrauma	Yes	65	0	0	0
	No	64			
Physical abuse	Yes	22	0	0	0
	No	107			
Sexual abuse	Yes	10	0	0	1
	No	119			cg03051003^e^
Alcohol	Yes	32	0	0	0
	No	97			
Cannabis	Yes	65	0	0	0
	No	64			

# number; *VASS* = violent aggression severity score

^a^comparisons with age, medication and cell counts as covariates

^b^CpG site in *TRAPPC11* gene 5’UTR, not previously associated

^c^CpG site in *RARA* gene (body), previously associated with tobacco smoking [56]

^d^total amount of migration-associated CpG sites at threshold 10^−6^ amounts to 90 CpG sites [5 (<10^−8^)+12(<10^−7^)+73(<10^−6^)]

^e^CpG site not previously associated (intergenic)

## Discussion

The present work was initiated based on the observation in a schizophrenia cohort that accumulation of environmental risk factors before adulthood promotes the likelihood of later forensic hospitalization, interpreted as indicator of violent aggression. This interpretation and the effect of risk accumulation were consolidated using direct scoring of aggression over lifetime or, as aggression proxies, forensic hospitalization and conviction for battery, sexual assault, manslaughter or murder, or respective psychopathology measures in 4 independent schizophrenia cohorts and 2 general population samples. Importantly, our data support the concept of a disease-independent development of violent aggression in subjects exposed to multiple pre-adult environmental risk factors.

Whereas a vast amount of literature on single environmental risk factors reports consequences for abnormal behavior and mental illness, publications on pre-adult risk accumulation are scarce and mostly based on closely interrelated social/familial risk factors. Also, risk and consequence are often not clearly defined. Studies including larger, comprehensively characterized datasets and replication samples do not exist. The present work is the first to provide sound evidence, based on 6 separate cohorts, of a disease-independent relationship between accumulation of multifaceted pre-adult environmental hits and violent aggression. The overall societal damage is enormous, and we note that mentally ill individuals who re-enter the community from prison are even more at risk for unemployment, homelessness, and criminal recidivism [37]. These results should encourage better precautionary measures, including intensified research on protective factors which is still underrepresented [2, 38–40].

In the psychosociological literature, the so-called externalizing behavior in childhood includes hostile and aggressive physical behavior toward others, impulsivity, hyperactivity, and noncompliance with limit-setting [41, 42]. The respective risk factors are all highly plausible, yet often theoretical, and derived from 4 broad domains: child risk factors (e.g., adverse temperament, genetic and gender risk), sociocultural risks (e.g., poverty, stressful life events), parenting and caregiving (e.g., conflict and violence at home, physical abuse), and children’s peer experiences (e.g., instable relationships, social rejection). A full model of the development of conduct problems has been suggested to include at least these 4 domains [41, 43, 44]. The risk factors analyzed in the present study are perhaps somewhat clearer defined but partially related to and overlapping across these domains. Urbanicity, migration, cannabis and alcohol reflect sociocultural input but also peer experience, and physical or sexual abuse belong to the parenting/caregiver aspect.

Certainly, there are many more, still undiscovered risk and numerous protective factors, potentially explaining why ‘only’ 40–50% of high-risk individuals in our schizophrenia samples fulfill criteria of violent aggression. We note that this study does not include genetic data analysis or correction for any genetic impact. The genetic influence on aggression, however, may be of considerable relevance for the individual [45–49], even though highly heterogeneous as for essentially all behavioral traits. Heritability of aggression, estimated from twin studies, reaches > 60% [50, 51]. In fact, 50% of individuals with violent aggression upon pre-adult risk accumulation in the present study means another 50% without detectable aggression. This consistent finding across samples likely indicates that genetic predisposition is prerequisite for whichever behavioral consequence. Individuals without genetic predisposition and/or with more protective factors (genetic and environmental) may not react with violent aggression to accumulated environmental risk. Importantly, the obvious gender effect may be a matter of degree rather than of pattern. In fact, the etiology of externalizing behavior problems is similar for girls and boys [41, 52], as is the consequence of risk accumulation in the present study for males and females.

The risk factors of the sociological domains seem to be stable predictors over time, to some degree interchangeable, pointing to many pathways leading to the same outcome (principle of equifinality) [41]. The interchangeability is highly interesting also with respect to potential biological mechanisms. It appears that any of the here investigated hits alone, independent of its kind, can be compensated for but that higher risk load increases the probability of violent aggression. Also for that reason, we are weighing risk fators equally in the present study. This could theoretically create some bias. However, to be able to estimate the true effect size of each specific factor separately on violent aggression and subsequently weigh all factors in a more proper way, much larger samples sizes would be needed that are presently not available anywhere in the world.

In contrast to the marginal influence of genome-wide association data on mental disease in GRAS [18, 53], the accumulated environmental impact on development of violent aggression is huge, reflected by odds ratios of > 10. When striking at a vulnerable time of brain development, namely around/before puberty, the environmental input may ‘non-specifically’ affect any predisposed individual. The hypothetical biological mechanisms underlying this accumulation effect in humans may range from alterations in neuroendocrine and neurotransmitter systems, neuronal/synaptic plasticity and neurogenesis to changes in the adaptive immune system and interference with developmental myelination, affecting brain connectivity and network function [9, 10, 54, 55].

Our approach to detect methylation changes in blood using an epigenome-wide association scan was unsuccessful despite matched extreme group comparison, likely due to the small sample size (although in the suggested range [32]), and perhaps the etiological/pathogenetic complexity of accumulated risks. Changes in brain, not accessible here for analysis, can certainly not be excluded. Interestingly, however, *HDAC1* mRNA levels in PBMC of male extreme group subjects were increased in the high-risk compared to the low-risk group. This finding confirms peripheral *HDAC1* mRNA levels as a more robust readout of epigenetic alterations in relatively small sample sizes [36], as compared to specific methylation sites in epigenome-wide association scans or even in candidate genes. To gain further mechanistic insight and thereby develop - in addition to prevention measures - novel individualized treatment concepts [36], animal studies modeling risk accumulation seem unavoidable.

To conclude, this study should motivate sociopolitical actions, aiming at identifying individuals-at-risk and improving precautionary measures. Effective violence prevention strategies start early and include family-focused and school-based programs [2, 16, 38]. Additional risk factors, interchangeable in their long-term consequences, like urbanicity, migration, and substance abuse, should be increasingly considered. Health care providers are essential for all of these prevention concepts. More research on protective factors and resilience should be launched. Animal studies need to be supported that model risk accumulation for mechanistic insight into brain alterations leading to aggression, and for developing new treatment approaches, also those targeting reversal of epigenetic alterations. As a novel concept, scientific efforts on *‘phenotyping of the environment’* [11] should be promoted to achieve more fundamental risk estimation and more effective prevention in the future.

## References

[1] McDonald C, Murray RM (2000). Early and late environmental risk factors for schizophrenia. Brain Res Rev.

[2] Raine A (2002). Biosocial studies of antisocial and violent behavior in children and adults: a review. J Abnorm Child Psychol.

[3] Read J, van Os J, Morrison AP, Ross CA (2005). Childhood trauma, psychosis and schizophrenia: a literature review with theoretical and clinical implications. Acta Psychiatr Scand.

[4] van Os J, Kenis G, Rutten BPF (2010). The environment and schizophrenia. Nature.

[5] Brown AS (2011). The environment and susceptibility to schizophrenia. Prog Neurobiol.

[6] Lederbogen F, Kirsch P, Haddad L, Streit F, Tost H, Schuch P (2011). City living and urban upbringing affect neural social stress processing in humans. Nature.

[7] Wortzel HS, Arciniegas DB (2013). A Forensic neuropsychiatric approach to traumatic brain injury, aggression, and suicide. J Am Acad Psychiatry.

[8] Orlovska S, Pedersen MS, Benros ME, Mortensen PB, Agerbo E, Nordentoft M (2014). Head injury as risk factor for psychiatric disorders: A Nationwide Register-based follow-up study of 113,906 persons with headinjury. Am J Psychiat.

[9] McEwen BS, Nasca C, Gray JD (2016). Stress effects on neuronal structure: hippocampus, amygdala, and prefrontal cortex. Neuropsychopharmacology.

[10] Nemeroff CB (2016). Paradise lost: the neurobiological and clinical consequences of child abuse and neglect. Neuron.

[11] Ehrenreich H (2017). The impact of environment on abnormal behavior and mental disease. EMBO Rep.

[12] Giancola PR. Alcohol-related aggression during the college years: Theories, risk factors and policy implications. J Stud Alcohol. 2002;14:129–39. 10.15288/jsas.2002.s14.129 12022718

[13] Heinz AJ, Beck A, Meyer-Lindenberg A, Sterzer P, Heinz A (2011). Cognitive and neurobiological mechanisms of alcohol-related aggression. Nat Rev Neurosci.

[14] Large M, Sharma S, Compton MT, Slade T, Nielssen O (2011). Cannabis Use and Earlier Onset of Psychosis. Arch Gen Psychiat.

[15] Walsh E, Gilvarry C, Samele C, Harvey K, Manley C, Tattan T (2004). Predicting violence in schizophrenia: a prospective study. Schizophr Res.

[16] Fazel S, Gulati G, Linsell L, Geddes JR, Grann M (2009). Schizophrenia and violence: systematic review and meta-analysis. PLoS Med.

[17] Sariaslan A, Lichtenstein P, Larsson H, Fazel S (2016). Triggers for violent criminality in patients with psychotic disorders. JAMA Psychiatry.

[18] Stepniak B, Papiol S, Hammer C, Ramin A, Everts S, Hennig L (2014). Accumulated environmental risk determining age at schizophrenia onset: a deep phenotyping-based study. Lancet Psychiatry.

[19] Begemann M, Grube S, Papiol S, Malzahn D, Krampe H, Ribbe K (2010). Modification of cognitive performance in schizophrenia by complexin 2 gene polymorphisms. Arch Gen Psychiat.

[20] Ribbe K, Friedrichs H, Begemann M, Grube S, Papiol S, Kastner A (2010). The cross-sectional GRAS sample: a comprehensive phenotypical data collection of schizophrenic patients. BMC Psychiatry.

[21] Alemany S, Moya J, Ibanez MI, Villa H, Mezquita L, Ortet G (2016). Research Letter: Childhood trauma and the rs1360780 SNP of FKBP5 gene in psychosis: a replication in two general population samples. Psychol Med.

[22] Ortet G, Ibanez MI, Moya J, Villa H, Viruela A, Mezquita L (2012). Assessing the five factors of personality in adolescents: the junior version of the Spanish NEO-PI-R. Assessment.

[23] Ribbe K, Ackermann V, Schwitulla J, Begemann M, Papiol S, Grube S (2011). Prediction of the risk of comorbid alcoholism in schizophrenia by interaction of common genetic variants in the corticotropin-releasing factor system. Arch Gen Psychiat.

[24] Bernstein DP, Stein JA, Newcomb MD, Walker E, Pogge D, Ahluvalia T (2003). Development and validation of a brief screening version of the Childhood Trauma Questionnaire. Child Abus Negl.

[25] Babor T, DeLaFuentes J, Saunders J, Grant M. The alcohol use disorders identification test: guidelines for use in primary health care. Vol. PSA. World Health Organization; Geneva, Switzerland, 1992. pp. 1–30.

[26] Levenson MR, Kiehl KA, Fitzpatrick CM (1995). Assessing psychopathic attributes in a noninstitutionalized population. J Pers Soc Psychol.

[27] Aluja A, Rossier J, Garcia LF, Angleitner A, Kuhlman M, Zuckerman M (2006). A cross-cultural shortened form of the ZKPQ (ZKPQ-50-cc) adapted to English, French, German, and Spanish languages. Pers Indiv Differ.

[28] Aryee MJ, Jaffe AE, Corrada-Bravo H, Ladd-Acosta C, Feinberg AP, Hansen KD (2014). Minfi: a flexible and comprehensive Bioconductor package for the analysis of Infinium DNA methylation microarrays. Bioinformatics.

[29] Ritchie ME, Phipson B, Wu D, Hu Y, Law CW, Shi W (2015). limma powers differential expression analyses for RNA-sequencing and microarray studies. Nucleic Acids Res.

[30] Jaffe AE, Irizarry RA (2014). Accounting for cellular heterogeneity is critical in epigenome-wide association studies. Genome Biol.

[31] Galanter JM, Gignoux CR, Oh SS, Torgerson D, Pino-Yanes M, Thakur N (2017). Differential methylation between ethnic sub-groups reflects the effect of genetic ancestry and environmental exposures. Elife.

[32] Tsai PC, Bell JT (2015). Power and sample size estimation for epigenome-wide association scans to detect differential DNA methylation. Int J Epidemiol.

[33] Waltes R, Chiocchetti AG, Freitag CM (2016). The neurobiological basis of human aggression: a review on genetic and epigenetic mechanisms. Am J Med Genet B Neuropsychiatr Genet.

[34] Gunter TD, Vaughn MG, Philibert RA (2010). Behavioral genetics in antisocial spectrum disorders and psychopathy: a review of the recent literature. Behav Sci Law.

[35] Kolla NJ, Matthews B, Wilson AA, Houle S, Bagby RM, Links P (2015). Lower Monoamine Oxidase-A Total Distribution Volume in Impulsive and Violent Male Offenders with Antisocial Personality Disorder and High Psychopathic Traits: An [(11)C] Harmine Positron Emission Tomography Study. Neuropsychopharmacology.

[36] Bahari-Javan S, Varbanov H, Halder R, Benito E, Kaurani L, Burkhardt S (2017). HDAC1 links early life stress to schizophrenia-like phenotypes. Proc Natl Acad Sci USA.

[37] Swanson J (2016). Mental illness, release from prison, and social context. JAMA.

[38] Sumner SA, Mercy JA, Dahlberg LL, Hillis SD, Klevens J, Houry D (2015). Violence in the United States: status, challenges, and opportunities. JAMA.

[39] Stoddard SA, Whiteside L, Zimmerman MA, Cunningham RM, Chermack ST, Walton MA (2013). The Relationship Between Cumulative Risk and Promotive Factors and Violent Behavior Among Urban Adolescents. Am J Commun Psychol.

[40] van der Laan AM, Veenstra R, Bogaerts S, Verhulst FC, Ormel J (2010). Serious, minor, and non-delinquents in early adolescence: the impact of cumulative risk and promotive factors. The TRAILS Study. J Abnorm Child Psychol.

[41] Deater-Deckard K, Dodge KA, Bates JE, Pettit GS (1998). Multiple risk factors in the development of externalizing behavior problems: Group and individual differences. Dev Psychopathol.

[42] Mcmahon RJ (1994). Diagnosis, assessment, and treatment of externalizing problems in children - the role of longitudinal data. J Consult Clin Psych.

[43] Herrenkohl TI, Maguin E, Hill KG, Hawkins JD, Abbott RD, Catalano RF (2000). Developmental risk factors for youth violence. J Adolesc Health.

[44] Loeber R, Pardini D, Homish DL, Wei EH, Crawford AM, Farrington DP (2005). The prediction of violence and homicide in young men. J Consult Clin Psych.

[45] Mason DA, Frick PJ (1994). The Heritability of Antisocial-Behavior - a Metaanalysis of Twin and Adoption Studies. J Psychopathol Behav Assess.

[46] Mednick SA, Gabrielli WF, Hutchings B (1984). Genetic influences in criminal convictions: evidence from an adoption cohort. Science.

[47] Coccaro EF, Bergeman CS, Kavoussi RJ, Seroczynski AD (1997). Heritability of aggression and irritability: a twin study of the Buss-Durkee aggression scales in adult male subjects. Biol Psychiatry.

[48] Miles DR, Carey G (1997). Genetic and environmental architecture of human aggression. J Pers Soc Psychol.

[49] Rhee SH, Waldman ID (2002). Genetic and environmental influences on antisocial behavior: A meta-analysis of twin and adoption studies. Psychol Bull.

[50] DiLalla LF, Gottesman II (1991). Biological and genetic contributors to violence--Widom’s untold tale. Psychol Bull.

[51] Plomin R, DeFries JC, Knopik VS, Neiderhiser J (2013). Behavioral genetics.

[52] Zoccolillo M (1993). Gender and the development of conduct disorder. Dev Psychopathol.

[53] Ehrenreich H, Mitjans M, Van der Auwera S, Centeno TP, Begemann M, Grabe HJ (2016). OTTO: a new strategy to extract mental disease-relevant combinations of GWAS hits from individuals. Mol Psychiatry.

[54] Korosi A, Naninck EFG, Oomen CA, Schouten M, Krugers H, Fitzsimons C (2012). Early-life stress mediated modulation of adult neurogenesis and behavior. Behav Brain Res.

[55] Nave KA, Ehrenreich H (2014). Myelination and oligodendrocyte functions in psychiatric diseases. JAMA Psychiatry.

[56] Su D, Wang X, Campbell MR, Porter DK, Pittman GS, Bennett BD (2016). Distinct epigenetic effects of tobacco smoking in whole blood and among leukocyte subtypes. PLoS ONE.

